# Extraction of Sub-microscopic Ca Fluxes from Blurred and Noisy Fluorescent Indicator Images with a Detailed Model Fitting Approach

**DOI:** 10.1371/journal.pcbi.1002931

**Published:** 2013-02-28

**Authors:** Cherrie H. T. Kong, Derek R. Laver, Mark B. Cannell

**Affiliations:** 1 Department of Physiology and Pharmacology, University of Bristol, Bristol, United Kingdom; 2 Department of Physiology, University of Auckland, Auckland, New Zealand; 3 School of Biomedical Sciences and Pharmacy, University of Newcastle and HMRI, Callaghan, New South Wales, Australia; University of Virginia, United States of America

## Abstract

The release of Ca from intracellular stores is key to cardiac muscle function; however, the molecular control of intracellular Ca release remains unclear. Depletion of the intracellular Ca store (sarcoplasmic reticulum, SR) may play an important role, but the ability to measure local SR Ca with fluorescent Ca indicators is limited by the microscope optical resolution and properties of the indicator. This leads to an uncertain degree of spatio-temporal blurring, which is not easily corrected (by deconvolution methods) due to the low signal-to-noise ratio of the recorded signals. In this study, a 3D computer model was constructed to calculate local Ca fluxes and consequent dye signals, which were then blurred by a measured microscope point spread function. Parameter fitting was employed to adjust a release basis function until the model output fitted recorded (2D) Ca spark data. This ‘forward method’ allowed us to obtain estimates of the time-course of Ca release flux and depletion within the sub-microscopic local SR associated with a number of Ca sparks. While variability in focal position relative to Ca spark sites causes more out-of-focus events to have smaller calculated fluxes (and less SR depletion), the average SR depletion was to 20±10% (s.d.) of the resting level. This focus problem implies that the actual SR depletion is likely to be larger and the five largest depletions analyzed were to 8±6% of the resting level. This profound depletion limits SR release flux during a Ca spark, which peaked at 8±3 pA and declined with a half time of 7±2 ms. By comparison, RyR open probability declined more slowly, suggesting release termination is dominated by neither SR Ca depletion nor intrinsic RyR gating, but results from an interaction of these processes.

## Author Summary

Calcium levels inside myocytes regulate the heart's force of contraction. Calcium is released from the primary intracellular store called the sarcoplasmic reticulum. Calcium release was directly observed as ‘calcium sparks’ using fluorescent calcium indicators inside the cell. More recently, calcium levels inside the store have been measured as calcium ‘blinks’. These suggest that some depletion of store calcium occurs during cell excitation; however, the actual extent of depletion is made uncertain by the complex sarcoplasmic reticulum shape, dye saturation and optical properties of the microscope. While previous studies have assumed idealized microscope properties, we measured microscope blurring and applied it to a computer model of calcium movements inside the cell. In this model, calcium release was adjusted to match the simulated blurred calcium signals to experimental results. The calculations show that the depth of local sarcoplasmic reticulum calcium depletion is much greater than inferred from calcium blinks and that the time-course of calcium release is affected by this depletion. An estimate for the time-course of gating of the ion channels that regulate calcium release inside the cell was also calculated. We suggest that the time-course of SR Ca release arises from a complex interaction of Ca depletion and channel gating.

## Introduction

During cardiac excitation-contraction coupling, calcium (Ca) is released from the sarcoplasmic reticulum (SR) through ryanodine receptors (RyRs), which are concentrated in the junctional regions of the SR (jSR). Ca release occurs due to ‘calcium-induced calcium release’ (CICR) [1], wherein Ca efflux from the jSR produces a rapid, local increase in Ca in the cytoplasm, which can be observed with fluorescent Ca indicators as a ‘Ca spark’ [2]. The corresponding Ca depletion in the junctional and wider SR has been detected as a ‘Ca blink’ [3]. The SR Ca signal is made possible by a protocol that favors indicator loading into the SR [4] and have shown that during a Ca spark, a ∼40% decrease in local SR [Ca] appears to occur [3], [5]. The fundamental insight provided by these and other biophysical approaches have led to local control theories [6] for the regulation of SR Ca release, however, detailed understanding of CICR has been elusive due to uncertainties in the amplitude and time-course of RyR release flux and the associated changes in local Ca concentrations near the RyRs. In particular, identification of the mechanism(s) responsible for termination of the inherently regenerative CICR mechanism has been especially problematic [7]. Nevertheless, integrating facets of known Ca handling systems has provided useful insight into the interplay of Ca metabolism with excitability (*e.g.* [8]–[12]).

Consideration of the sub-microscopic volume of the jSR immediately suggests that the Fluo-5N signal must under-estimate true jSR Ca depletion [3], [13]. This problem arises from size of the confocal point spread function (PSF), which encompasses both elements of the jSR and adjacent network SR. In the latter, [Ca] is high and is likely to change with a different time-course to that in the jSR. It should be noted that this situation is different during Ca spark recordings because the cytoplasmic signal is larger than the jSR volume and the confocal PSF integrates signal from regions that have low [Ca]. The potential seriousness of the blurring problem has led us to analyze the problem by using computational methods, combined with measurements of actual microscope blurring to extract the likely depth SR Ca depletion, which is central to understanding the termination of SR Ca release during Ca sparks.

In principle, the problem of microscopic blurring might be reduced by deconvolution of the recorded signal with the microscope PSF. However, Ca blink signals are extremely noisy which renders this approach (essentially) unusable (a problem frequently observed in inverse solutions). Our approach is to create a computer model that captures the general geometry underlying local SR Ca release, blur the simulated fluorescent Ca signals, then refine the underlying flux parameters by iteration until experimental records are reproduced. This ‘forward method’ for analyzing SR Ca release was introduced by Soeller and Cannell [10]. We show that local jSR depletion is likely to be heavily under-estimated by Fluo-5N signals. With estimates of jSR depletion and release flux, we were also able to obtain the first estimates of the time-course of RyR gating at the junction (from recorded data) and show that the decline of release flux is not simply due RyR closure. This result should have important implications for models of CICR termination.

## Methods

### Cell preparation

This study was performed in strict accordance with the recommendations in the Guide for the Care and Use of Laboratory Animals of the National Institutes of Health. The protocol used (R649) was approved by the University of Auckland Animal Ethics Committee. All surgery was performed under sodium pentobarbital anesthesia (140 mg/kg, i.p.), and every effort was made to minimize suffering.

Single cardiac ventricular myocytes were obtained by enzymatic dissociation of Langendorff-perfused hearts from male Wistar rats (∼250 g), as previously described [14]. Cells were incubated with 5 µM Fluo-4 acetoxymethyl ester (Invitrogen, California, U.S.A.) for 25 min by adding 2.5 µL of a 2 mM stock (2.5% Pluronic® F-127 in dimethyl sulfoxide, Invitrogen and Sigma-Aldrich, respectively) to 1 mL of cell suspension. Following incubation, the dye loading solution was replaced with perfusion buffer, which was a modified Tyrode's solution (pH = 7.4, in mM): NaCl 140, KCl 4, MgCl_2_ 1, HEPES 10, D-glucose 10 and CaCl_2_ 1. Experiments were carried out at room temperature.

### Line-scanning confocal microscopy

Imaging was performed with a LSM 710 (Zeiss, Oberkochen, Germany) system. Resolution of the line-scan images was higher than usually performed (*e.g.* [2], [15]) at ∼0.35 ms/line and 0.083 µm/pixel, with the scan line placed along the cell.

The actual microscope PSF was measured by imaging 100 nm yellow-green Fluorospheres (Invitrogen). Beads resting on the glass cover-slip of the perfusion bath and on top of live myocytes were imaged and analyzed to determine the ideal microscope PSF and the maximum PSF distortion by spherical aberration (note that the cell does not have the same refractive index as the immersion medium). This process was performed for both a water- (40×, 1.1 numerical aperture) and an oil- (63×, 1.4 numerical aperture) immersion objective. The latter is typical of objectives used in most other Ca spark and Ca blink studies (*e.g.* [2], [3], [5], [15]).

### Model parameters and diffusion equations

The computer model equations were solved numerically by FACSIMILE (Flow and Chemistry Simulator, U.K. Atomic Energy Authority, 1987). FACSIMILE solutions were then analyzed by custom programs written in Interactive Data Language (IDL v6.3, ITT Visual Information Solutions, Colorado, U.S.A.). Solutions were fit to experimental data by adjusting the peak and time-course of RyR permeability using a non-linear least-squares method [16]. The accuracy of this fitting method was tested using synthetic datasets with Poisson noise and reliable convergence was found in all cases.

The computer model consisted of both cytosolic and SR compartments and was based on spherical geometry. The computational volume had a radius of 4 µm, which was divided into 40 elements (1≤i≤39), where the center-most element (i = 0) contained the jSR with reflective boundary conditions at the edge of the computational space. The diffusional volumes of the cytosol and SR were 60% and 3.2% of total cell volume [17] respectively, except for the first element, where SR volume was set to 8 aL to represent the jSR [3].

The equation to be solved is
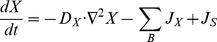
(1)where D_X_ is the diffusion coefficient for diffusible species *X*, *J_X_* the reactive flux for all buffers *B* and *J_S_* the source (and sinks) of that species. The Laplacian in one dimension for spherical geometry was solved by a conventional finite difference scheme. Reactive fluxes were described by the general ODE:
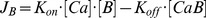
(2)where B and CaB are the ligand-free and Ca-bound forms of the buffer, respectively. All rate constants, concentrations and diffusion coefficients of buffers are given in Table 1. FACSIMILE automatically scaled the calculated flux across compartment boundaries to take account of the different compartment volumes. The general approach has been described previously [11].

**Table 1:** Computer model buffers: concentrations, diffusion coefficients and binding kinetics.

x	[x]	D	K_on_	K_off_	K_D_
	(µM)	(10^−8^ dm^2^/s)	(/µM/s)	(/s)	(µM)
**Cytosol**					
**Ca_free_**	0.1	3.0	-	-	-
**ATP**	4000	1.5	13.64	30000	2200
**CaM**	36 (4 sites)	0.45	100	31	0.31
**Fluo-4**	50	0.75	307	276.7	0.9
**Fluo-5F**	100	0.75	307	451.9	1.47
**TnC_I,hi_**	140 (2 sites)	-	100	7.1	0.071
**TnC_II,lo_**	70	-	125	500	4
**SL_m_**	86	-	125	1625	13
**SR_m_**	47	-	115	100	0.87
**SR**					
**Ca_free_**	1000	*	-	-	-
**CSQ**	120000	-	100	600000	600
**Fluo-5N**	50	*	307	122956	400

For details, see Methods.

### SR Ca release function

SR Ca release flux (µM/ms) was determined by the [Ca] gradient between the jSR and junctional space and a flexible basis release function, modified from [10]:
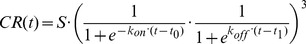
(3)where the constants *k*_on_ and *k*_off_ controlled the rising and decay phases, respectively, *S* a scaling factor for magnitude and *t*_0,1_ were time shifts. Only these five parameters were adjusted by least squares minimization of model output to recorded Ca sparks, all other parameters were as given in Table 1. The sensitivity of the model to chosen parameters is shown in the Supporting Information.

The calculated release flux (*I_spark_*) was used to calculate *n·P_O_* of the RyRs by:
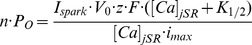
(4)where the maximum unitary RyR channel current (i_max_) and half maximal conductance (K_1/2_) were set to 2 pA and 2 mM [Ca]_jSR_, respectively [18]. These constants give a single channel current (i_RyR_) of ∼0.6 pA when [Ca]_jSR_ is 1 mM. V_0_ was the volume of the first cytosolic element (4.18 aL), z is the valence of Ca and F is Faraday's constant.

### Buffering and transport

**Figure 1:**
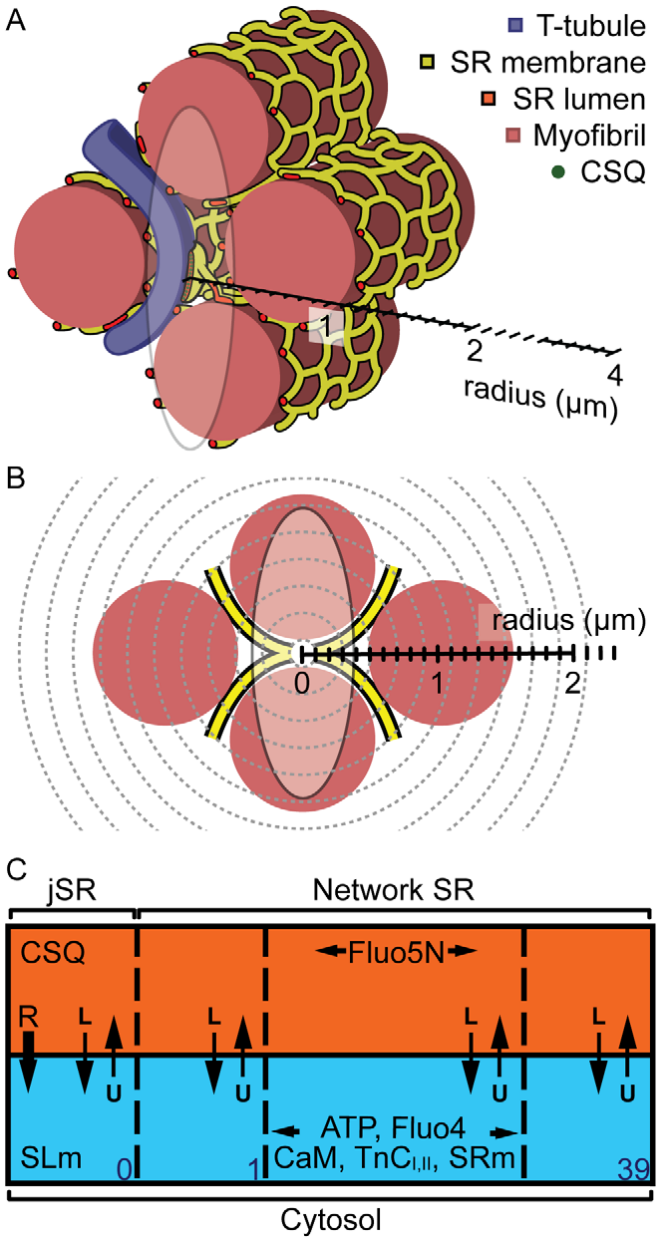
Computer model geometry. (**A**) shows a region of a cardiac myocyte and presents the size of a typical junction relative to a confocal PSF and the computer model. A transverse tubule (purple) extends in between myofilaments (pink) that are wrapped by network of SR tubules (yellow surface, red lumen). A flattened disc of SR wraps around the T-tubule to form a Ca release junction. The size of a typical confocal PSF is shown by an ellipse in x-z orientation, at 2·FWHM. (**B**) shows the transverse, stylised view of (A), where the SR can be seen as an ‘X’ shape that curves around the myofibrils. The jSR is shown as a white circle and assumed to be in the centre of the PSF (opaque ellipse). The spherical mesh of the computer model is also shown (grey dashed lines), with the radius at 4 µm, which should be sufficiently large to capture a Ca spark without boundary effects. (**C**) shows a simplified diagram of the computer model elements (0≤i≤39) and the two compartments: the cytosol (blue) and SR (orange). The locations of mobile and immobile Ca buffers are shown. See text and Table 1 for details.

As shown in Fig. 1B, the SR contained Fluo-5F, while only the jSR compartment contained calsequestrin (CSQ) and was able to release Ca (“R”), which entered the first cytosolic element. The rate of SR uptake (“U”) were set so that when [Ca]_i_ was transiently increased to 10 µM, return to rest occurred in a half-time of ∼160 ms:
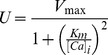
(5)where V_max_ was 300 µM/s and K_m_ was 0.3 µM [17]. The leak flux (“L”) was set so that [Ca]_i_ at rest was 100 nM [19]. Cytosolic buffers included ATP, calmodulin (CaM), Fluo-4, troponin-C (both high and low affinity sites, TnC_I_ and TnC_II_, respectively) and SR membrane binding sites (SR_m_). The junctional space also included sarcolemmal membrane binding sites (SL_m_) and excluded Fluo-4 [10]. [Mg] was set to 1 mM in all compartments [20].

The diffusion coefficient for ATP (D_ATP_) at 24°C was chosen as an intermediate between the estimates obtained by two methods: (1) If D_ATP_ is 1.4×10^−8^ dm^2^/s at 16°C [12] and the Q_10_ of D_ATP_ is 1.4 [21], then D_ATP_ would be 1.9×10^−8^ dm^2^/s at 24°C. (2) Since the molar mass of ATP is 507.18 g/mol, assuming a globular structure would allow the Stoke-Einstein equation to predict that D_ATP_ would be 1.4×10^−8^ dm^2^/s. The total concentration of ATP was set to 4 mM [12], the majority of which was bound to Mg at rest (K_on,Mg_ = 3.3×10^−4^/µM/s, K_off,Mg_ = 3/s, [22]). The on and off rates of Fluo-4 were also estimated from experimental data obtained at 16°C [23] using a Q_10_ of 2. Dissociation constants of Fluo-4 and Fluo-5F were obtained from various in vitro calibrations [24], [25]. D_CaM_ was estimated from its Stoke's radius [26]. TnC contained three binding sites for Ca, where the high affinity sites (TnC_I,hi_) could also bind Mg (K_on,Mg_ = 0.03/µM/s, K_off,Mg_ = 1.11/s, [27], [28]). TnC_II,lo_ bound exclusively to Ca [22], [27], [28]. Ca binding sites were also present on the sarcolemmal [29] and SR [30] membranes. Resting SR free [Ca] was 1 mM [31], [32]. The jSR contained CSQ binding sites equivalent to 120 mM, due to a binding capacity of 30–40 Ca per molecule of CSQ [33].

The diffusion coefficient for Ca (D_Ca_) in the SR was set to a value smaller than that in the cytosol to include the effect of tortuosity and Ca binding sites within the SR. Considering the SR network as a sheet with staggered holes as diffusion barriers, the tortuosity factor can be estimated from the ratio between the size of and distance between the ‘holes’. Since SR tubules are ∼40 nm in diameter and the ‘holes’ in the SR network between adjacent tubules are ∼160 nm wide [34], the diffusion coefficient would be reduced by 70% [35]. The binding of Ca to buffers in the SR lumen will also reduce the rate of Ca diffusion in proportion to the number of binding sites present. It is thought that ∼60% of Ca buffers in the network SR is SERCA [36], with ∼1.7 mmol/L_SR_ of SERCA in rat [37]. Assuming each SERCA molecule has two binding sites for Ca and free [Ca]_SR_ is 1 mM and the other buffers have 1∶1 reactions with Ca, then the concentration of binding sites would be ∼2.8 mmol/L_SR_ and diffusion would be reduced by ∼65%. Thus, the overall effect of tortuosity and Ca binding is a ∼90% reduction in effective D_Ca_. In addition, the local network SR Ca flux into a jSR is likely to be further reduced due to reduced connectivity (by only one or two tubules, [3]). This was accounted for by further reducing the diffusion coefficient between the last network SR element and the jSR element. If there were only one tubule of 40 nm diameter connecting these elements, then the rate of diffusion between them would be reduced by ∼95% [38]. Increased confidence in the value used was provided by the ability of the model to reproduce the time course of Ca blink recovery (*e.g.* [5]). The diffusion coefficient of Fluo-5N within the SR was assumed to be 5 times smaller than for Fluo-4 in the cytoplasm [39]. Using different intra-SR Ca diffusion coefficients had relatively small effects on the simulated Ca spark (Supporting Information Fig. S1, red crosses). For example, when D_Ca,SR_ was increased 10-fold, peak fluorescence and FWHM increased by less than ∼10%, while the time-course was prolonged (time to peak increased to ∼13 ms from 7.5 ms and time to half decay ∼doubled). On the other hand, altering the intra-SR Ca diffusion coefficient had large effects on Ca spark restitution (not shown) and Ca blink recovery. With the value(s) used here, experimental results were reasonably reproduced (see below).

### Incorporation of microscope optical blurring

Two three-dimensional (3D) Gaussian functions were used to simulate the PSFs recorded at the coverslip and on tops of cells. For the coverslip PSF, the Gaussian function had a FWHM of 0.25 µm in the focal plane (x, y) and 0.6 µm along the optical axis (z). For cell top PSF, the Gaussian function had a FWHM of 0.35 µm in x, y and 1.2 µm in z.

The Ca-bound Fluo-4 signal has to be spatially blurred at each computational time-point to simulate an experimentally recorded Ca spark in a line-scan image. This raises a computational problem because efficient solution of the reaction diffusion equations needs to exploit symmetry (where possible) to reduce the dimensionality, while the PSFs are asymmetric. Furthermore, during parameter fitting, very large numbers of simulation runs may be carried out, so computational efficiency is highly desirable and suggests that limiting the problem to a spherical coordinate system might be advantageous. While re-gridding the solution at each time point to Cartesian coordinates for convolution by the PSF would be possible, this time-consuming operation was avoided by transforming sets of PSF weights from Cartesian to radial ordinates, each corresponding to a particular position of the PSF convolved with a spherical shell. This radial PSF could be pre-computed (to save time) and the convolution performed in FACSIMILE. This entire process was repeated at each time-point to generate the blurred signal, which was equivalent to a confocal line-scan image. The complete line-scan image was then used during least squares fitting (see above).

The optical distortion of the Ca-bound Fluo-5N signal during the formation of a Ca blink is not the same that for a Ca spark due to different geometry of the SR with respect to the confocal PSF. The volume of the signal of interest is much smaller and the signal is likely to be contaminated by signal from the network SR. To simulate this situation, the SR signal was distributed as an extruded “X” shaped spatial region centered on the jSR to mimic the SR network wrapping around the myofilaments (see Fig. 1A and Fig. 1B).

### Parameter sensitivity

To test the sensitivity of the model to chosen parameters, parameters were independently altered and the simulated Ca^2+^ spark compared to a ‘standard’ using transport parameters given in Table 1 which: reached a peak F/F_0_ of 1.8 in 7.5 ms, had a time to half decay of 17 ms and a FWHM of 1.2 µm. The effects of altering [ATP] in the cytosol, [CSQ] in the jSR, jSR volume, [Fluo-4], S, k_on_ and k_off_ are shown in Fig. S1. From these analyses, we can predict how the release flux time-course would have to be altered to match experiments; for example an increase in peak amplitude would require correspondingly larger flux (and deeper SR Ca depletion). An increase in time to peak would require a corresponding decrease in speed of decay of the release flux. Note that increasing the volume of the jSR (red triangles) had a similar effect to increasing [CSQ] (see [40]) since this also increases the amount of Ca available for release. The effect of altering the shape and amplitude of the Ca release function (i.e. S, k_on_, k_off_), as occurred during curve-fitting, are also shown. The effect of altering SR Ca diffusion on Ca spark fits is illustrated in Supporting Fig. S2.

## Results

### Effect of myoplasm on the confocal PSF

**Figure 2:**
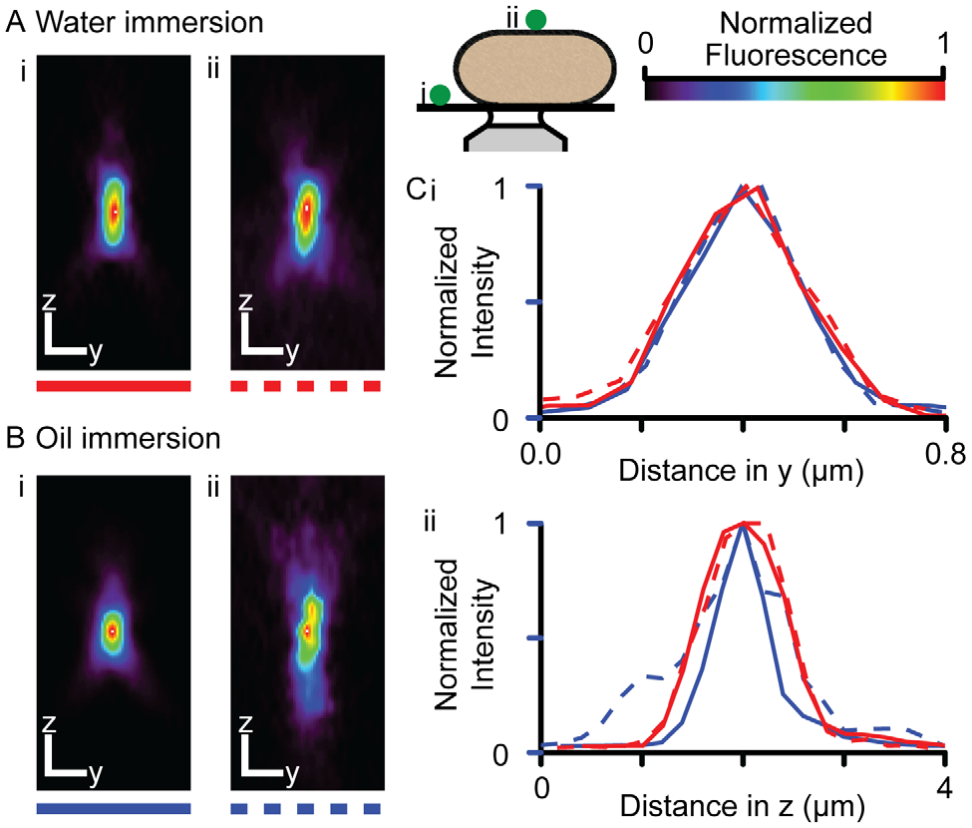
The effect of live myocytes on the confocal PSF. Examples of PSFs measured using (**A**) water and (**B**) oil immersion objectives on the coverslip (**i**) and on top of a live cardiac myocyte (**ii**) bathed in 1 mM Ca-Tyrode's solution are shown (see inset). Scale bars indicate 0.5 µm. (**C**) The intensity profiles across the peak intensity in (**i**) y and (**ii**) z are shown. The effect of the refractive index mismatch across the myocyte was more pronounced when using an oil immersion objective.

Images of yellow-green beads located on the coverslip (Fig. 2i) of a perfusion chamber and on top of live myocytes (Fig. 2ii) for both water- (Fig. 2A) and oil- (Fig. 2B) immersion objectives are shown. It is notable the recorded PSF is clearly distorted along the optical axis, an effect which we attribute to refractive index mismatch(es). A summary of PSF dimensions is given in Table 2, and it is clear that the distortion in z is most pronounced when using the oil-immersion objective (as might be expected), where the FWHM of the PSF was almost doubled. It is clear from these data that assumption of an idealized (*i.e.* diffraction limited) PSF during Ca spark and Ca blink recording would be erroneous.

**Table 2:** Measurement of confocal point spread functions during live cell imaging.

	FWHM (µm)			
	x	y	z	n
**Water immersion**				
Coverslip	0.38±0.03	0.35±0.02	1.7±0.2	6
Cell	0.34±003	0.36±0.03	1.5±0.1	5
**Oil immersion**				
Coverslip	0.29±0.01	0.22±0.02	0.61±0.04	4
Cell	0.32±0.01	0.32±0.02	1.19±0.06	5

Mean FWHM of PSFs on the coverslip and on top of live myocytes with oil and water immersion objectives (one S.E.M. shown). The focal plane is described by x and y, while the optical axis is described by z.

### Optical blurring of a Ca spark signal

**Figure 3:**
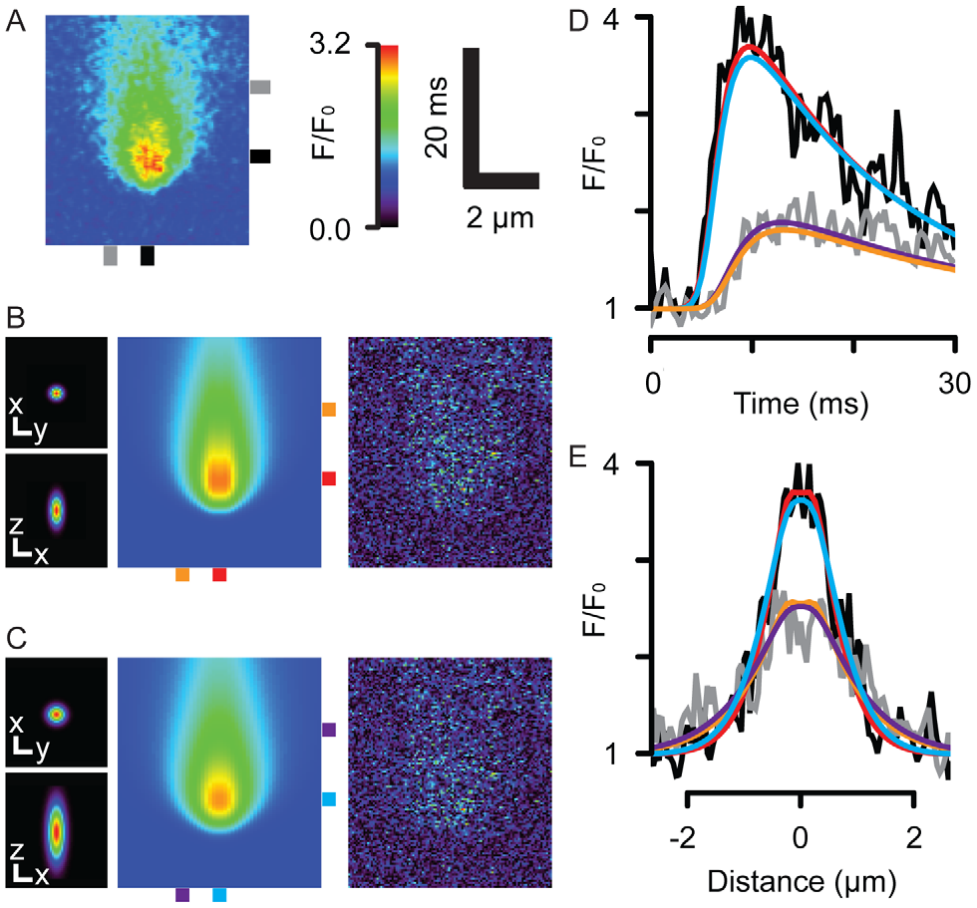
Simulation and fit of a Ca spark with on-coverslip and on-cell PSFs. (**A**) shows a line-scan image of a measured Ca spark. From left to right, (**B**) shows the on-coverslip PSF in x-y (top) and x-z (bottom) view (scale bars show 0.5 µm), the simulated Ca spark and absolute difference between the simulated and recorded events. The mean of the difference image was −0.06. (**C**) shows a similar dataset, but for an on-cell PSF, where the mean of the difference image was −0.07. The goodness of fit can be appreciated in the time and distance profiles are shown in (**D**) and (**E**), respectively, colored by the marks beside the Ca spark images.

Two 3D Gaussian profiles based on our PSF measurements were used to simulate the effect of PSFs that are on the coverslip or on top of cells. A high signal-to-noise spontaneous Ca spark is shown in Fig. 3A and was used as the data set for parameter-fitting. Fig. 3B shows the coverslip PSF (shown in in x-y and x-z views) and the Ca spark generated by the computer model. The image on the right shows the absolute difference between the simulated and recorded events, showing a good fit with no systematic residuals. Fig. 3C shows the results of using the cell-top PSF, where the model could also fit the recorded event with a larger release flux (see below). The measured (black lines) and fitted time (Fig. 3D) and spatial (Fig. 3E) profiles of the Ca sparks are also shown, colored according to the markers shown in Fig. 3A–C. These profiles also show that the fitted and measured events are in reasonable agreement.

**Figure 4:**
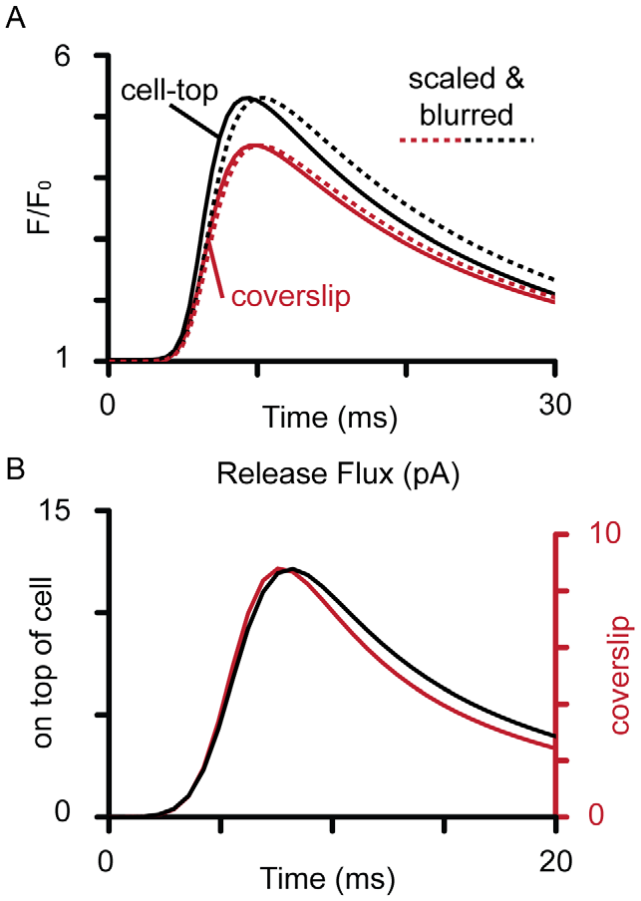
Ca-Fluo-4 and release flux associated with Ca spark. The (**A**) time profiles of the unblurred calculated Ca-Fluo-4 signals for Ca sparks at the top of the cell (black) and near the coverslip (red). The dashed lines show the corresponding simulated Ca sparks (blurred Ca-Fluo-4 signals) scaled to the amplitudes of the un-blurred signals to allow comparison of the time-courses. (**B**) shows the release flux required to produce the fitted simulated Ca sparks shown in Fig. 3 and 4A, where a larger current was required when optical blurring was more severe (*i.e.* if the Ca spark had originated from near the cell top).

The un-blurred Fluo-4 dye signal at the center of the Ca spark is shown in Fig. 4A, as calculated if the recorded Ca spark occurred at the cell-top (black lines) or near the coverslip (red lines), respectively. The dashed lines correspond to the blurred dye signals, where they been scaled to the amplitude of the un-blurred signals to allow comparison of the effect of blurring on time-course. Despite being in-focus, optical blurring in both cases caused the recorded Ca spark to be slower than that of the underlying signal. For a Ca spark that originated at the cell-top, the time of peak fluorescence was increased by ∼1 ms. This effect was smaller when the Ca spark was at the coverslip. The FWHM of the Ca spark was doubled due to blurring (not shown). The fluxes that were required to produce the same recorded Ca spark (Fig. 3) were different due from using the two different PSFs (Fig. 4B). When the cell-top PSF was used, the calculated peak release flux was ∼11.8 pA, while when the smaller, coverslip PSF was used, only ∼8.7 pA was required to produce the blurred Ca spark. Since these values represent two extreme cases for Ca sparks recorded in-focus, but from different locations within a cell, it is likely that an average Ca spark would be associated with an intermediate peak current (*e.g.* ∼10 pA). Note that the different PSFs did not markedly change the time-course of the calculated release flux.

### Optical blurring of a Ca blink signal

**Figure 5:**
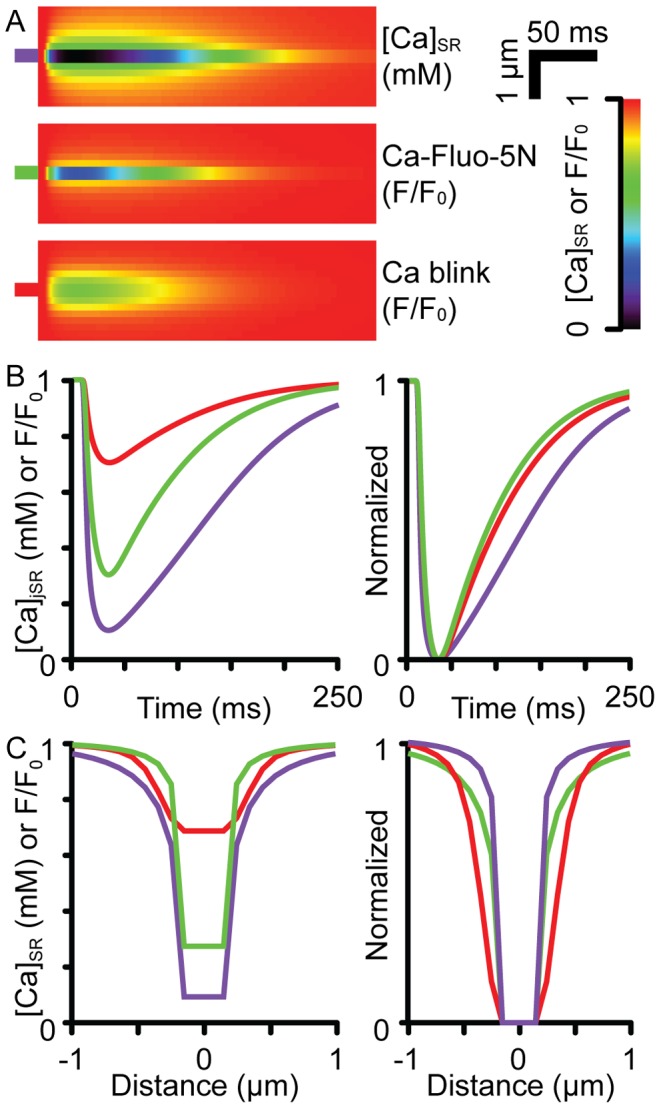
Ca blinks and [Ca]_SR_ signals. (**A**) shows the simulated line-scan images of [Ca]_SR_ (purple), Ca-Fluo-5N (green) and Ca blink (blurred Ca-Fluo-5N, red) signals when the Ca sparks were simulated with an cell-top PSF. Left panels in (**B**) and (**C**) show the time and spatial profiles through the minimum Ca blink intensity, respectively. The right panels show scaled versions of the profiles.

Fig. 5A shows the simulated line-scan images of [Ca]_SR_, Ca-Fluo-5N and the Ca blink (optically blurred Ca-Fluo-5N) signals generated from using the cell-top PSF marked by purple, green and red bars, respectively. Depending on the degree of optical blurring determined by the orientation of the network SR relative to the optical axis (see Fig. 1B), Ca blink depletions ranged between 25–35%, which are within the range of values reported in other studies [3], [5], [39]. Importantly, the underlying non-blurred Ca-Fluo-5N signal showed much more extensive depletion compared to the corresponding Ca blink. For example, the Ca-Fluo-5N signal had decreased by ∼70% at its minimum, more than double that suggested by the blurred signal. Additionally, Ca-Fluo-5N also under-reported the underlying depletion of [Ca]_SR_, which was actually ∼90%. Overall, dye kinetics and optical blurring caused a near 3-fold under-estimation of the true extent of [Ca]_SR_ depletion. Dye kinetics also had an effect on the ability of Ca blink signals to correctly report the time-course of [Ca]_SR_ changes (right panel, Fig. 5B). Though there were only small distortions to the time to minimum, the time taken for [Ca]_SR_ recovery was under-estimated by Ca-Fluo-5N by ∼40 ms and which was slightly lengthened by optical blurring. Overall, this led to a ∼35% under-estimation of [Ca]_SR_ recovery time.

### Local SR Ca release flux and depletion

**Figure 6:**
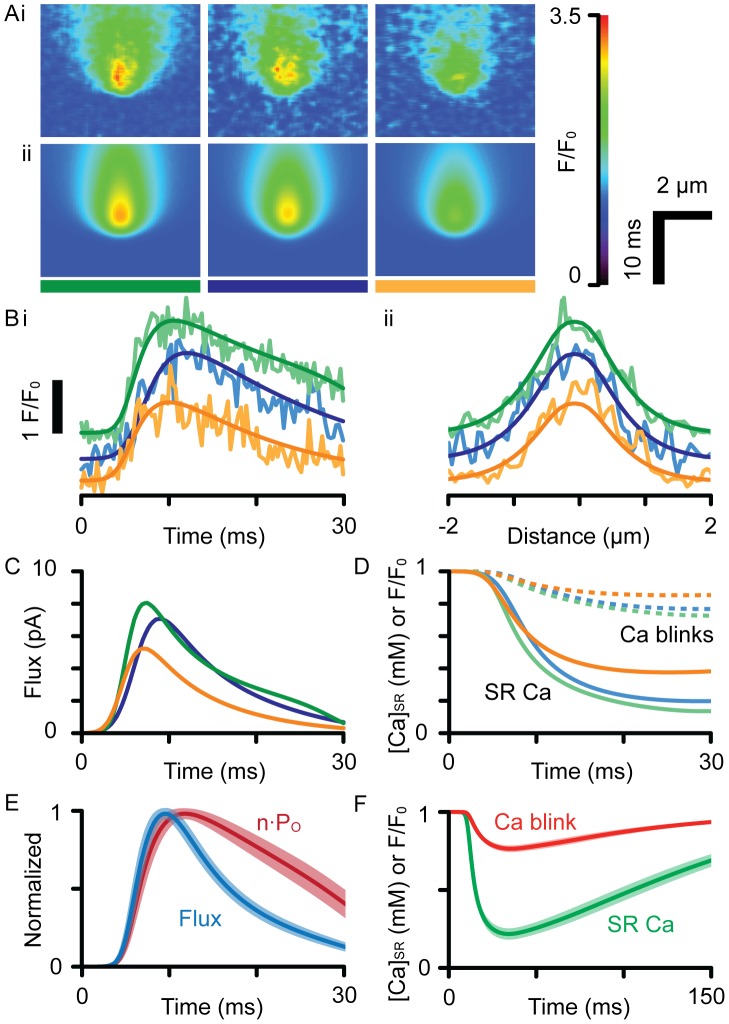
Analysis of recorded Ca sparks. (**A**) shows 3 examples of (**i**) recorded and (**ii**) simulated Ca sparks analyzed with a cell-top PSF. These events were chosen for their high signal-to-noise ratio and high amplitude, which suggests they are in-focus. (**B**) shows the (**i**) time and (**ii**) spatial profiles of the recorded and fitted (smooth lines) datasets, color-coded by the bars shown in (A). The events have been offset for clarity. (**C**) shows the flux responsible for each event, which was used to calculate the time-course of n·P_O_. (**D**) shows the time-course of [Ca]_SR_ in the junction (solid lines), overlaid with the corresponding Ca blink signals (dashed lines). (**E**) shows the time-courses of release flux and n·P_O_ averaged from fitting a population of Ca sparks (n = 14), where the shaded regions show one S.E.M. For all events, RyRs were open longer than release flux duration, which were 24.0±13 vs. 11.2±2.2 ms, respectively, when measured from the start to the time at half maximal decay. (**F**) shows the corresponding average [Ca]_jSR_ and Ca blink time-courses with the shaded areas showing one S.E.M.

Fig. 6A and B show further examples of model fits to a number of experimentally recorded Ca sparks with different amplitudes and time-courses. The ability of the model to fit this range of Ca sparks is notable. The computed Ca release functions are shown in Fig. 6C, where peak flux occurred before the peak of the Ca spark. The calculated SR Ca signals in Fig. 6D show that [Ca]_SR_ depletion was large compared to their associated Ca blink signals (dashed lines). In addition, the duration of the computed release flux was always shorter than the duration of the fitted permeability function, where peak flux always occurred before RyR open probability had even begun to decline (Fig. 6E), showing jSR depletion plays an important role in reducing release flux prior to closure of the RyR channels. The mean peak flux was 7.9±2.9 pA (s.d.) for Ca sparks with maximum F/F_0_ of 3.0±0.6. Panel F shows average Ca blinks compared to average Ca-Fluo-5N and [Ca]_SR_ signals, consistent with the trends described in Fig. 5. The average (blurred) Ca blink signal was ΔF/F_0_ = 0.24±0.098 (s.d.), compared to the corresponding SR depletion to an average minimum of 210±130 µM.

## Discussion

### Effect of microscope blurring on Ca sparks

The model presented here shows that the Ca spark signal is distorted by the confocal PSF even when it is in-focus, so that its amplitude was approximately halved and width doubled. This is consistent with calculations reported previously, albeit with different PSF and a more narrow modeled spark FWHM of ∼1 µm [30], [41]. The calculated peak release flux is dependent on the degree of spatial blurring, but our estimate is in reasonable accord with previous models [10], [41]. This concordance might not have been expected when many models do not reproduce the spatio-temporal properties of recorded Ca sparks. This can be explained by the peak flux being the primary determinant of the peak change in fluorescence (which all models fit well), which is largely dominated by flux amplitude. Initial analyses of Ca sparks (with a mean F/F_0_ of ∼2.0) in early studies gave a flux estimate of 4 pA for 10 ms [2] and it is notable that initially estimated integrated flux, ∼40 pA.ms has not changed a great deal in subsequent analyses (*e.g.* see [29], [41]). This is due to the need to preserve total Ca release (from integrated peak fluorescence) during a Ca spark. In these simulations, a Ca spark of F/F_0_ ∼2.7 yields a flux integral of ∼40 pA.ms which is in good agreement with previous estimates, given the somewhat higher peak F/F_0_ recorded here.

### Blurring in Ca blink signals

The distortion introduced by microscope blurring was more pronounced for Ca blinks compared to Ca sparks. This problem arises from: (1) the width of the PSF (as is the case for Ca sparks), as well as (2) the spatially restricted nature of the underlying signal source, (3) the non-linear dye response; and (4) the high [Ca] in the extended network SR. The overall effect of these factors was that the Ca blink amplitude must under-estimate the extent of jSR depletion even when the microscope is focused on a z-line and at a junction. Our average estimated [Ca]_jSR_ signal fell to ∼20% of the resting level, which is much lower than the decline to estimated previously [5], [39], even after including a correction for fractional jSR volume [3]. Since many Ca sparks are out of focus, this value should represent an upper limit for the actual SR depletion. Taking the brightest 5 Ca sparks as representative of in-focus events gave a jSR depletion to 8±6% (mean ± s.d.) of the resting level.

### Local SR Ca depletion

The actual depletion in [Ca]_jSR_ is certainly larger than directly inferred from the fluorescence signal due to the non-linear dye response, which will always cause the dye signal to underestimate true average Ca change (*i.e.* dF/dCa≪1). Furthermore, the network SR supplies a high Ca signal that changes more slowly than the jSR. Although this component might be captured by a correction for relative volume (as noted by [3]), the extent of the correction is critically dependent on the spatial distribution of intra-SR gradients and the relative volume of network to jSR for a particular junction (*e.g.* ∼30% further under-estimation if the SR and myofilaments were rotated 90° relative to the PSF, Fig. 1B). That the model reproduces Ca blink signals after blurring indicates that the profound jSR depletion we predict is completely consistent with experimental data and shows that previous estimates of jSR Ca depletion are almost certainly too small.

The depth of jSR Ca depletion is affected by the rate at which the jSR is refilled by the network SR. If D_Ca_ in the SR is increased 10-fold, the time-course of recovery of Ca blinks is not reproduced (Fig. S2). Although such a change in SR D_Ca_ did not prevent fitting the Ca spark shown in Fig. 3, the corresponding Ca blink was small (ΔF/F_0_ ∼0.15, Fig. S2C, dotted lines) and recovered in 12.5 ms, which is much faster than any Ca blinks reported to date. Despite this much larger rate of diffusion within the SR, profound depletion of Ca in the jSR was still observed (∼60%, Fig. S2, solid lines) and the Ca blink was still spatially restricted (Fig. S2D).

An independent test of the calculations is provided by comparing the average SR Ca depletion that would occur during an evoked Ca transient or during a Ca wave. Assuming the unit of release includes the jSR and the network SR limited to a radius of ∼0.9 µm (from consideration of the distance from the release site to the middle of the sarcomere), the model predicts that the jSR (z-line) Ca depletion would be ∼3-times larger than that at the m-line. However, during synchronous release, the m-line becomes depleted more deeply as more CRUs draw Ca from it. Using our model, a first order solution to this problem is provided by assuming that each CRU is not affected by release from adjacent sites so that the depletion is given by restricting the SR volume to the equivalent volume that would serve each CRU. CRU's form an approximately hexagonal lattice with 0.7 µm between them in the z-plane and 1.8 µm along the cell axis [42]. Thus, the equivalent volume of the SR source Ca in the spherical model would have a radius of 0.52 µm, and the model predicts that the m-line would deplete to level much closer (90%) to that seen at the z-line. Therefore, if all CRU's are activated, the z- and m-line depletion signals are very similar (albeit with slightly different time-courses) and there would be (essentially) no detectable gradient along the sarcomere at typical Fluo-5N signal-to-noise ratios, as observed [13], [43].

### Effect of jSR depletion on the Ca release time course

The steep Ca gradient across the jSR membrane is reduced rapidly during a Ca spark (Fig. 6). The effect of this local jSR Ca depletion has a profound effect on the time-course of Ca release. As shown in Fig. 3–5, Ca release flux had decreased to half its peak value (at ∼5 ms after peak flux), the Ca gradient from the jSR to the cytosol had decreased to ∼64% of the value at the time of peak flux. The small difference between the flux and gradient reflects the minor contribution of changes in RyR n·P_O_ to the declining flux at this time, accounting for ∼12% of the total flux decline. Therefore, the decline in flux is not dominated by changes in RyR gating but rather the loss of driving force for Ca release at this time (Fig. 6E). The smaller decrease in n·P_O_ at this time may be explained by the RyRs operating in a near-saturated regime with regard to either jSR or cytoplasmic Ca control of RyR open time. Though not modeled here, it is highly likely that the reduction in release flux due to local Ca depletion will be transduced via the steep cytoplasmic Ca-dependence of RyR gating to reduce n·P_O_ and eventually stop CICR [44]. That reduced jSR [Ca] affects CRU gating has been explored by Sato and Bers [45], who concluded that SR depletion can prevent Ca spark initiation. This is in accord with the idea that the SR Ca depletion described here inhibits the regeneration inherent in CICR (and will therefore contribute to Ca spark termination).

### The confocal PSF during live cell Ca imaging

As shown in Fig. 2 and Table 2, the properties of a confocal PSF can deviate markedly from theoretical values when imaging occurs through a living cardiac myocyte. This is not simply due to spherical aberration (since this should be negligible with the water immersion lens) and the PSF is larger than the ∼0.2×0.7 µm expected for an oil-immersion lens focused into 10 µm of water (see also Table 20.2 in [46]). Visual inspection of the fine structure of the PSF suggests that other problems beyond simple spherical aberration exist: the bending of the PSF axis and structure in the field suggest that the cell is behaving as a complex phase object rather than as a simple body of fluid. In connection with this point, it is known that the cell surface is quite uneven (*e.g.* [47]) and the presence of myofilaments, mitochondria and even the nucleus (although the latter was avoided here) add complex structures that are barely resolved yet contribute to the phase shift of the marginal rays that are critical for tight PSF formation.

### Conclusions

This model accurately reproduced the reported spatial and temporal properties of Ca blinks and Ca sparks. The under-estimation of [Ca]_jSR_ depletion by Ca blink signals is somewhat larger than the results of a recent computer modeling study by Hake, *et al.* [9] would suggest. Although the latter study did not use experimental data sets nor microscope blurring (and slightly different parameters), the constraint of geometry and microscope performance on the accuracy of recorded signals is clear. The profound SR Ca depletion we calculate strongly affects SR release time-course and shows that the declining Ca flux during a Ca spark is not simply due to RyR closure. By fitting a model with realistic geometry to recorded data, we limit the effects of noise that would preclude inverting the equations for diffusion and buffering. It was notable that despite using an extensive dataset, all solutions converged toward similar time-courses for the release flux, suggesting an unexpected invariance of RyR gating behavior during SR release.

## Supporting Information

Figure S1 **Parameter sensitivity of Ca^2+^ spark model.** **(A)** Peak fluorescence, **(B)** FWHM, **(C)** time to peak and **(D)** time to half decay. The change in default parameters are normalized to their values given in Table 1. V_JSR_ is the volume of the jSR.
(TIF)

Figure S2 **Effect of increasing the rate of Ca diffusion in the SR on fitted Ca spark and Ca blink properties.** The Ca spark shown in Fig. 3 could be fit by least-squares minimization when D_Ca_ in the SR was decreased 10-fold. The data (grey lines) and fitted result (solid black lines) in time **(A)** and space **(B)** are shown, which show a reasonable fit, although the size of the residuals is larger than that shown in Fig. 3 (mean absolute difference = 0.07). The corresponding SR Ca signals in time **(C)** and space **(D)** are also shown. They include: [Ca]_SR_ (in mM, solid line), Ca-Fluo-5N (in F/F_0_, dashed line) and the blurred dye signal (Ca blink in F/F_0_, dotted lines).
(TIF)
