# Supplementary figures and images for: Extraction of Sub-microscopic Ca Fluxes from Blurred and Noisy Fluorescent Indicator Images with a Detailed Model Fitting Approach
Source: PLoS Comput Biol. 2013 Feb 28;9(2):e1002931. doi: 10.1371/journal.pcbi.1002931 (PMC3585382; doi:10.1371/journal.pcbi.1002931)

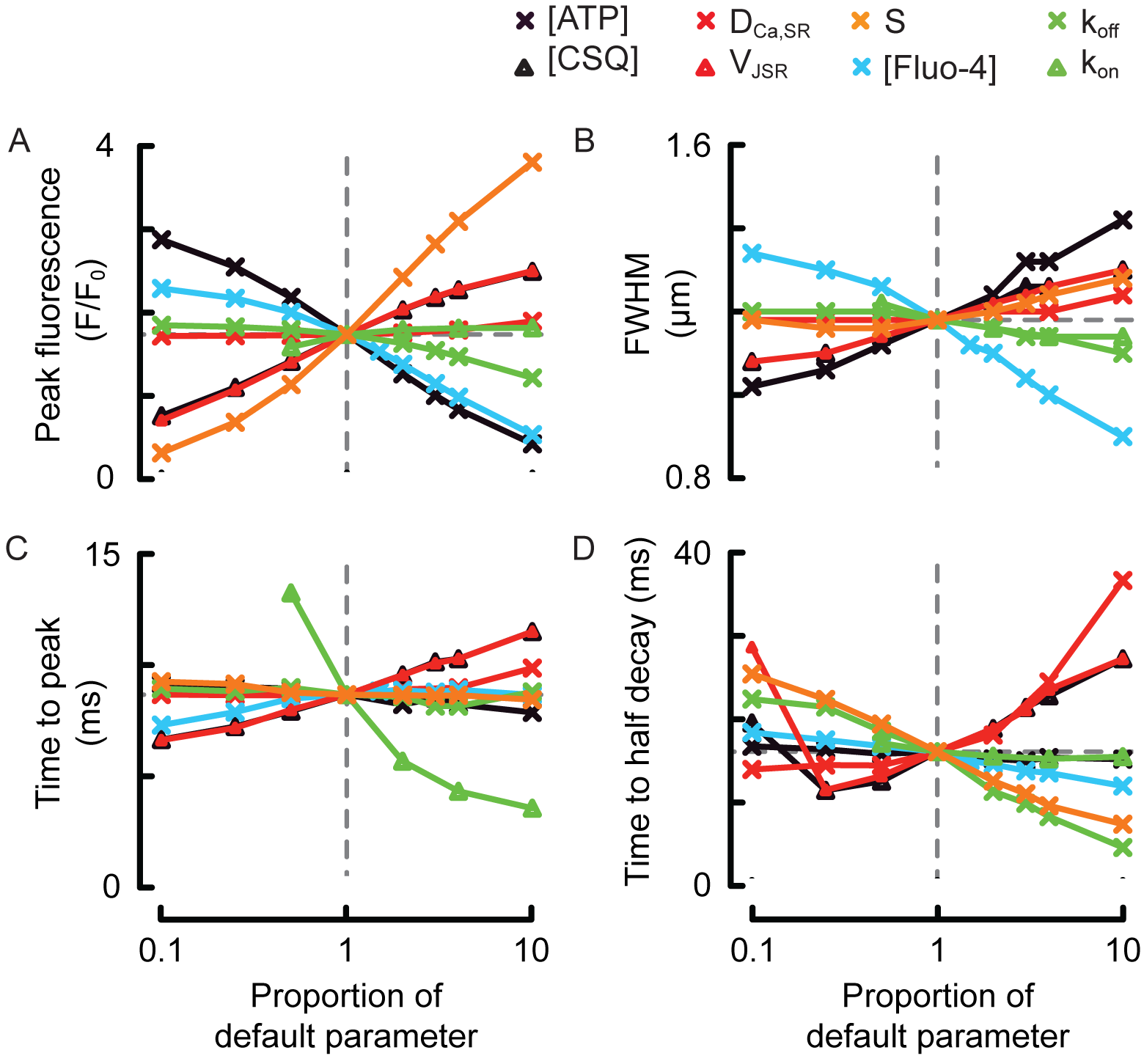

Supplement: Figure S1 — Parameter sensitivity of Ca2+ spark model. (A) Peak fluorescence, (B) FWHM, (C) time to peak and (D) time to half decay. The change in default parameters are normalized to their values given in Table 1. VJSR is the volume of the jSR. (TIF) [file pcbi.1002931.s001.tif]

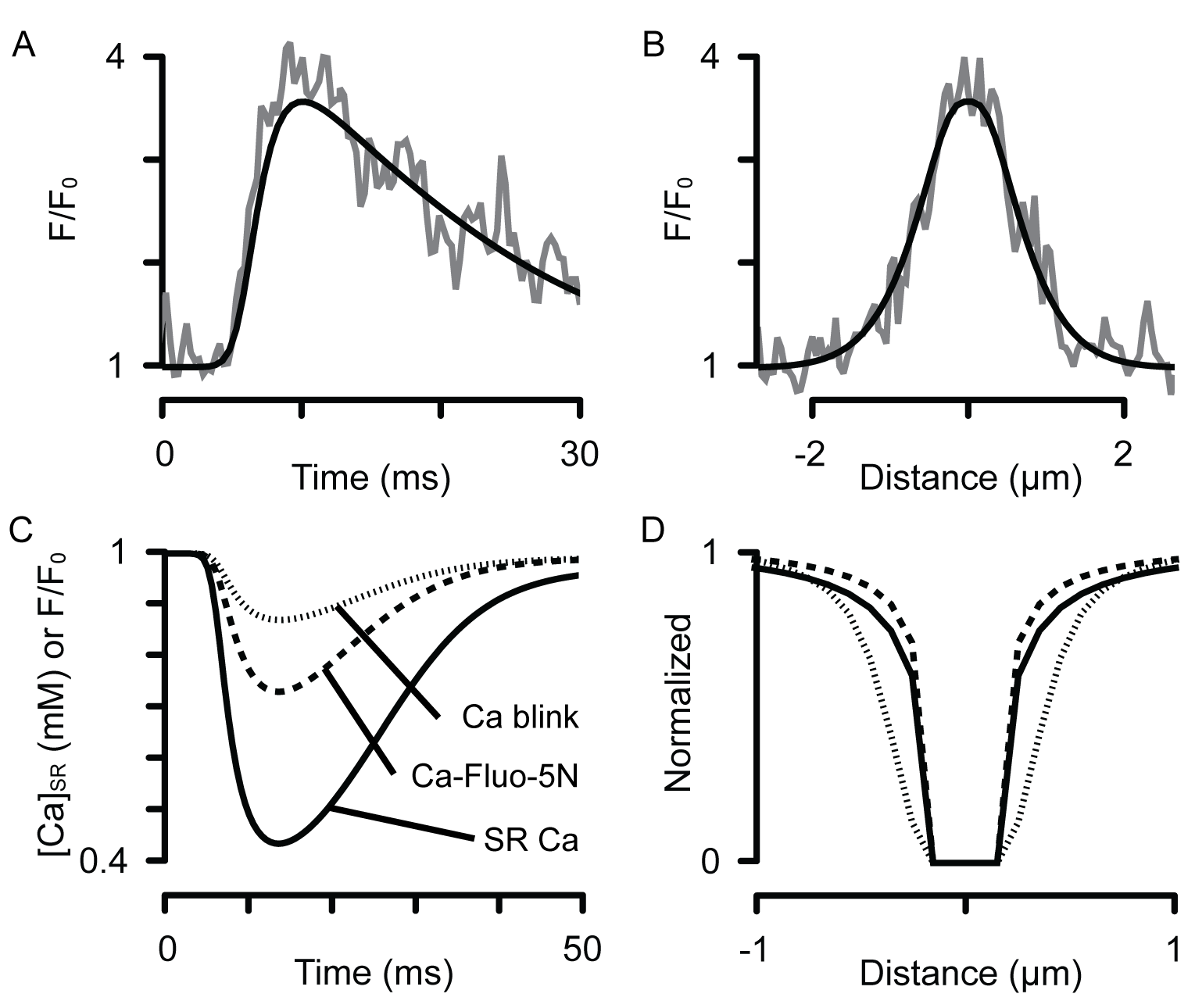

Supplement: Figure S2 — Effect of increasing the rate of Ca diffusion in the SR on fitted Ca spark and Ca blink properties. The Ca spark shown in Fig. 3 could be fit by least-squares minimization when DCa in the SR was decreased 10-fold. The data (grey lines) and fitted result (solid black lines) in time (A) and space (B) are shown, which show a reasonable fit, although the size of the residuals is larger than that shown in Fig. 3 (mean absolute difference = 0.07). The corresponding SR Ca signals in time (C) and space (D) are also shown. They include: [Ca]SR (in mM, solid line), Ca-Fluo-5N (in F/F0, dashed line) and the blurred dye signal (Ca blink in F/F0, dotted lines). (TIF) [file pcbi.1002931.s002.tif]
